# Siltuximab as a first-line therapy for idiopathic multicentric Castleman disease: a retrospective analysis based on the SiMuLa study of the Italian regional network

**DOI:** 10.3389/fonc.2026.1762473

**Published:** 2026-02-16

**Authors:** S. Pelliccia, A. Di Rocco, G. Palumbo, A. Zizzari, O. Annibali, E. Maiolo, A. Rago, G. Trapè, M. P. Bianchi, G. Pileggi, L. Filomeno, S. Hohaus, L. Rigacci, M. C. Cox, M. Martelli, G. La Verde, A. Di Napoli, A. Tafuri

**Affiliations:** 1 Hematology, Department of Clinical and Molecular Medicine, University Hospital Sant’Andrea – Sapienza, Rome, Italy; 2 Hematology, Department of Translational and Precision Medicine Univ Sapienza, Rome, Italy; 3 Hematology, Department of Biomedicine and Prevention, University Tor Vergata, Rome, Italy; 4 Research Unit of Hematology, Department of Medicine and Surgery, Università Campus Bio- Medico di Roma, Rome, Italy; 5 University Policlinico Gemelli Foundation-IRCCS, Catholic University of the Sacred Heart, Roma, Italy; 6 Hematology Unit, Azienda Sanitaria Locale (ASL) ROMA 1, Rome, Italy; 7 Hematology Unit, Ospedale Belcolle, Viterbo, Italy; 8 Pathology Unit, Sant’Andrea Hospital, La Sapienza University, Department of Clinical and Molecular Medicine, Sapienza University, Rome, Italy

**Keywords:** Castleman disease, interleukin-6, multicentric, real-world evidence, siltuximab

## Abstract

**Background:**

Idiopathic Multicentric Castleman Disease (iMCD) is a rare, heterogeneous lymphoproliferative disorder characterized by polyclonal lymphoid hyperplasia, systemic inflammatory symptoms, and generalized lymphadenopathy that can result in multiorgan dysfunction. The interleukin-6 (IL-6)–mediated hyperinflammatory state represents the key pathogenic mechanism of iMCD. Currently, first-line therapy is based on Siltuximab, with or without corticosteroids. Siltuximab (SYLVANT) is a chimeric monoclonal antibody that binds soluble human IL-6, forming stable, high-affinity complexes that neutralize its biological activity. Approximately 40–50% of patients fail to achieve a complete response during first-line Siltuximab therapy. This study aimed to retrospectively evaluate real-world outcomes and safety in patients with iMCD treated with first-line Siltuximab outside clinical trials across the Lazio region of Italy.

**Methods:**

Real-world data were retrospectively collected from patients with iMCD who received at least one dose of Siltuximab as first-line therapy (11 mg/kg every 3 weeks, until disease progression) between 2018 and March 2025 at Hematology Units across the Lazio region, Italy. Treatment responses were assessed according to Castleman Disease Collaborative Network (CDCN) criteria, based on biochemical, radiologic, and clinical parameters. Safety was evaluated using NCI-CTCAE version 5.0.

**Results:**

Fourteen patients were included. The median age was 54 years (range, 18–81), and the median treatment duration was 28 months (range, 3–55). The complete remission rate (CRR) was 29%, while the overall response rate (ORR) reached 86%. At data cutoff, 12 of 14 patients (85.7%) were alive. No infusion-related reactions occurred, and the overall safety profile was favorable.

**Conclusion:**

This preliminary real-world experience confirms that first-line Siltuximab is effective and well tolerated in patients with iMCD, achieving durable disease control in the majority of cases. These findings support the continued use of Siltuximab as the standard of care and underscore the importance of expanding real-world registries to better define the epidemiology and treatment outcomes of iMCD in Italy.

## Introduction

Idiopathic Multicentric Castleman Disease (iMCD) is a rare and potentially life-threatening lymphoproliferative disorder characterized by systemic inflammation, generalized lymphadenopathy, cytopenias, and multiorgan dysfunction (1). It belongs to the spectrum of Castleman Disease (CD), which is broadly classified into unicentric (UCD) and multicentric (MCD) forms (2–5). The idiopathic variant of MCD is defined by the absence of known etiologies—particularly human herpesvirus-8 (HHV-8), HIV infection, or monoclonal gammopathies—distinguishing it from HHV-8–associated MCD and POEMS-associated MCD. The incidence of iMCD is estimated at approximately 3–4 cases per million individuals per year, making it a rare condition with limited prospective data (6–8).

The pathophysiology of iMCD remains incompletely understood, but dysregulated cytokine signaling—especially involving interleukin-6 (IL-6)—plays a central role in driving both the systemic inflammatory response and the lymphoproliferative manifestations of the disease. Elevated IL-6 levels correlate with disease activity and underlie many of its hallmark features, including fever, fatigue, weight loss, anemia, hypoalbuminemia, and elevated C-reactive protein (CRP). Histopathologic findings in affected lymph nodes are heterogeneous, commonly displaying hypervascular, plasmacytic, or mixed features.

Diagnosing iMCD is challenging due to its overlap with autoimmune, infectious, and lymphomas (9–12). To improve diagnostic accuracy, the Castleman Disease Collaborative Network (CDCN) established evidence-based consensus criteria that require both characteristic histopathological features and a constellation of clinical and laboratory abnormalities, along with the exclusion of alternative diagnoses (13, 14).

Therapeutic approaches to iMCD have evolved substantially over the past decade. While corticosteroids and conventional immunosuppressants were historically employed with variable efficacy, targeting the IL-6 signaling pathway now represents the cornerstone of modern management. Siltuximab (SYLVANT), a chimeric monoclonal antibody that binds and neutralizes IL-6, is currently the only therapy approved by both the U.S. Food and Drug Administration (FDA) and the European Medicines Agency (EMA) for the treatment of iMCD. Clinical trials have demonstrated that Siltuximab induces durable symptomatic, biochemical, and radiologic responses in a substantial proportion of patients (15–18). Nevertheless, real-world data indicate that approximately 40–50% of patients do not achieve adequate disease control with first-line Siltuximab, and predictors of response remain poorly defined (19, 20).

Given the rarity of iMCD and the exclusion of many real-world patient populations from clinical trials, retrospective and observational data are essential to better understand treatment outcomes in routine clinical practice. Regional and national registries can provide valuable insights into disease presentation, response to therapy, safety, and long-term management strategies in unselected patient populations (21–26).

In this context, we conducted a retrospective observational multicenter analysis of patients diagnosed with iMCD and treated with first-line siltuximab across hematology centers in the Lazio region of Italy. The SiMuLa study (Siltuximab Multicentric Lazio) included all patients with idiopathic multicentric Castleman disease (iMCD) diagnosed according to the CDCN criteria who received at least one dose of siltuximab in routine clinical practice. The presence of a regional hematology network involving both hematologists and pathologists in the Lazio region enabled the harmonization of diagnostic and therapeutic pathways. The objective of this study was to evaluate the real-world effectiveness, safety, and tolerability of siltuximab outside controlled clinical trials, thereby contributing to a more comprehensive understanding of treatment outcomes in this rare disease.

## Methods

### Patient enrollment and diagnosis

This retrospective study enrolled 14 patients diagnosed with MCD treated with at least one dose of Siltuximab as in first line in accordance with clinical practice (11 mg/kg every 3 weeks, until disease progression), between January 2018 and March 2025 at the Hematology Units of Lazio region in Italy (University Hospital Sant’Andrea, University Hospital Umberto I, Catholic University S. Cuore, Tor Vergata University, Campus Biomedico University, Hematology Unit ASL-RM1 and Belcolle Hospital Viterbo). Eligible adults age >18 years with iMCD were diagnosed based on detailed patient history, physical examination, assessment of laboratory abnormalities, pathologic diagnosis, radiologic imaging, and histologically confirmed diagnosis from an excisional lymph node biopsy acquired before enrollment. For each patient, the following data were collected: baseline demographic characteristics (sex and age at diagnosis), clinical characteristics (comorbidities and number of involved lymph node regions), laboratory parameters, treatment exposure, clinical outcomes (treatment response), safety outcomes (adverse events), and follow-up data, including date of last follow-up, vital status at last follow-up, date of death, and cause of death. Patients included in the study were confirmed to be HIV seronegative and human herpesvirus-8 negative, as determined by a polymerase chain reaction test. Written informed consent was obtained from participants. The independent ethics committee approved the protocol (Rif. 7684 Prot. 0565/2024). The study was performed in accordance with the Declaration of Helsinki.

### Treatment and clinical outcomes

All enrolled patients received at least one dose of Siltuximab every three weeks (11 mg/kg), until treatment failure. Treatment failure was defined as the emergence of new disease-related symptoms with NCI-CTCAE grade ≥3, persistence of disease-related symptoms of grade ≥2 for more than three weeks, an increase in the ECOG performance score by more than one point, persistent symptoms for at least three weeks, or radiological progression. Treatment response was evaluated according to the Castleman Disease Collaborative Network (CDCN) consensus guidelines. Three composite endpoints were used to assess response: (A) four laboratory parameters of inflammatory response and organ function, including hemoglobin, CRP, albumin, and eGFR, with eGFR further subdivided into blood urea nitrogen and serum creatinine levels; (B) four critical clinical symptoms: fatigue, anorexia, fever, and weight change; and (C) lymph node size. Complete remission (CR) required normalization of clinical symptoms, biochemical markers, and lymph node response, assessed using the modified Cheson criteria. Partial remission (PR) was defined by a reduction of more than one NCI-CTCAE grade in clinical symptoms, over 50% improvement in all biochemical markers (though not returning to baseline), and PR in lymph node response. Stable disease (SD) was defined as the absence of CR, PR, or progressive disease (PD). Progressive disease (PD) was defined by a >50% worsening in any biochemical marker, the worsening of symptoms in two consecutive assessments, or a >25% increase in lymph node size. Response assessments were performed according to routine clinical practice, with non-uniform (opportunistic) timing, reflecting the real-world nature of the study. The overall response rate (ORR) was defined as the percentage of patients who achieved CR or PR.

### Statistical analyses

Continuous variables were summarized as median and range (or interquartile range, where appropriate). Categorical variables were reported as frequencies and percentages. Overall survival (OS) was defined as the time from the first dose of Siltuximab to death from any cause. Event-free survival (EFS) was defined as the time from initiation of Siltuximab to discontinuation, disease progression, relapse, or death from any cause, whichever occurred first. Patients without events were censored at the last follow-up. Kaplan–Meier survival analysis was performed to estimate OS and EFS, and log-rank tests were used for comparisons where applicable. Time from diagnosis to treatment initiation (TTT) was calculated as the number of days between the date of diagnosis and the first dose of Siltuximab. Statistical analyses were performed using SPSS version 28.0, with a two-sided p-value <0.05 considered statistically significant.

## Results

### Patient characteristics

Fourteen patients with idiopathic Multicentric Castleman Disease (iMCD) treated with first-line Siltuximab were included in this multicenter retrospective study. The median age at diagnosis was 54 years (range, 18–81), with a slight female predominance (8 females, 57%). Histopathological subtypes were plasmacytic in 5 patients (36%), mixed in 6 (43%), and hypervascular in 3 (21%), reflecting the heterogeneous histologic spectrum of iMCD.

At baseline, most patients (92.9%) had an Eastern Cooperative Oncology Group (ECOG) performance status <2, indicating preserved functional capacity. B symptoms (fever, night sweats, or weight loss) were present in 42.9% of patients. Laboratory evaluations revealed evidence of systemic inflammation and immune activation, with a median hemoglobin of 12.0 g/dL (range, 8.5–15.2), median C-reactive protein (CRP) of 8.7 mg/L (range, 0.1–157.3), median erythrocyte sedimentation rate (ESR) of 45 mm/h (range, 2–120), median albumin of 4.2 g/dL (range, 2.4–5.1), and median IgG of 1.6 g/dL (range, 1.01–16.3). Functional imaging with ^18F-FDG PET/CT showed a median SUVmax of 5.3 (range, 2.3–30.5). Baseline demographics and clinical characteristics are summarized in **Table 1**.

**Table 1:** Patient demographics and baseline clinical characteristics (n = 14).

Characteristic	Value
Median age, years (range)	54 (18–81)
Sex, n (%)	
Female	8 (57.1)
Male	6 (42.9)
Histopathological subtype, n (%)	
Hyaline vascular	3 (21.4)
Plasmacytic	5 (35.7)
Mixed	6 (42.8)
ECOG performance status ≥2, n (%)	
No	13 (92.9)
Yes	1 (7.1)
Presence of B symptoms, %	42.9
Median hemoglobin, g/dL (range)	12.1 (8.5–15.2)
Median C-reactive protein (CRP), mg/L (range)	8.7 (0.1–157.3)
Median erythrocyte sedimentation rate (ESR), mm/h (range)	45 (2–120)
Median albumin, g/dL (range)	4.2 (2.4–5.1)
Median IgG, g/dL (range)	1.6 (1.01–16.3)
Median PET SUVmax (range)	5.3 (2.3–30.5)

ECOG, Eastern Cooperative Oncology Group; CRP, C-reactive protein; ESR, erythrocyte sedimentation rate; SUVmax, maximum standardized uptake value.

### Treatment exposure

All patients received siltuximab at a standard dose of 11 mg/kg intravenously every three weeks. Concomitant corticosteroids were administered at treatment initiation in six patients (43%), with a median prednisone-equivalent dose of 25 mg die. During treatment with siltuximab, corticosteroids were successfully discontinued with a median time to withdrawal of 1.5 months (range, 1-12), while no patients required steroid re-initiation.

The median duration of siltuximab treatment was 28 months (range, 3–55). The longest-treated patient received siltuximab for 78 cycles. The median follow-up was 13 months (range, 1–49). Patients received a median of 37 cycles of siltuximab, and the median time from diagnosis to treatment initiation (TTT) was 93 days (range, 15–173).

### Efficacy outcomes

Overall, progression and event free survival were assessed starting from the first siltuximab infusion (T0) and evaluated during routine clinical follow-up visits, reflecting real-world practice. The overall response rate (ORR) was 86%, with complete remission (CR) achieved in 29% of patients, partial remission (PR) in 57%, and stable disease in 14%. Among responders, improvements in constitutional symptoms and laboratory abnormalities were typically observed within the first two months of therapy. The median time to best response was 6 months (range, 1–31).

**Figure 1:**
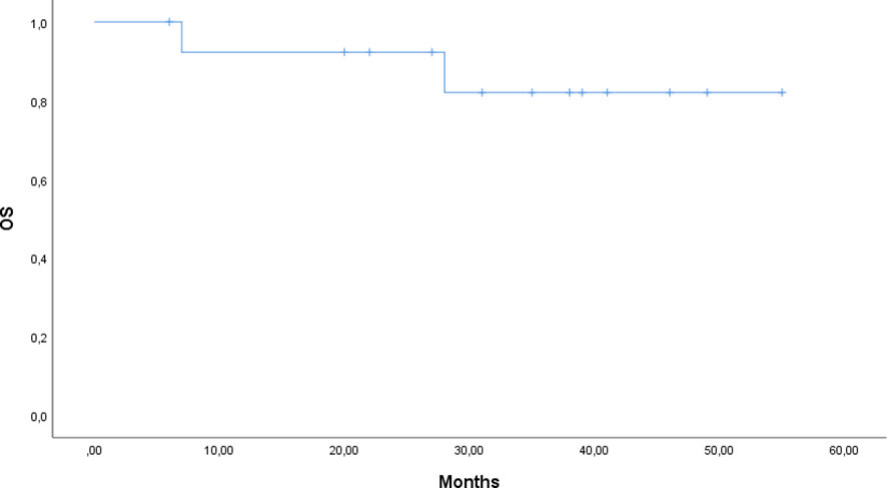
Kaplan–Meier curve of overall survival (OS) for patients treated with siltuximab.

**Figure 2:**
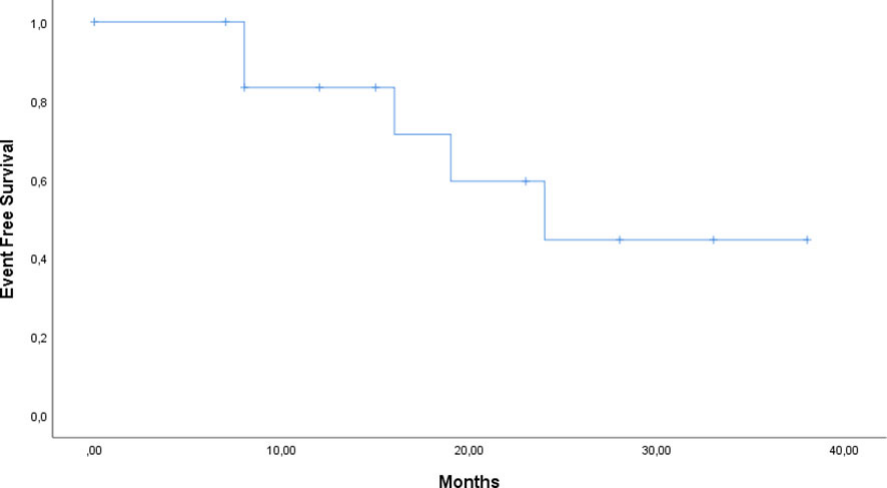
Kaplan–Meier curve of Event Free Survival (EFS) for patients treated with siltuximab.

At data cutoff (31^st^ March 2025), 12 of 14 patients (85.7%) were alive: one patient died from a lung infection caused by a multidrug-resistant pathogen, and a second patient died due to a lymphoproliferative neoplasm (Diffuse Large B-cell Lymphoma). Three patients discontinued Siltuximab at 18, 20, 41 months respectively, following the diagnosis of another malignancy. A last subject discontinued due to the onset of an adverse event and one due to disease progression. The median duration of response was not reached, reflecting durable benefit with continued therapy. Event-free survival (EFS) was 64.3%, and overall survival (OS) was 85.7% (**Figures 1**, **2**).

### Safety and tolerability

Siltuximab was generally well tolerated. No infusion-related reactions were reported during the study period. Grade ≥2 adverse events occurred in 3 patients (21%), including G3 hypertension, G2 transaminitis, and pneumonia in two patients of Grade 3 and 5 respectively. No patient experienced infusion-related reactions and the treatment was otherwise well tolerated. No hematologic toxicities, autoimmune events, or treatment-related deaths were observed. The overall safety profile was consistent with that reported in clinical trials, and no new or unexpected adverse events emerged in this real-world cohort.

### Summary

This real-world, multicenter experience confirms that first-line Siltuximab is effective and manageable in patients with iMCD, achieving durable disease control in nearly all treated patients. The high ORR, low toxicity burden, and sustained survival observed across multiple centers in the Lazio region emphasize the reproducibility of Siltuximab’s clinical benefits outside of controlled trials.

## Discussion

This multicenter retrospective study provides real-world evidence on the activity and safety of first-line Siltuximab in patients with idiopathic Multicentric Castleman Disease (iMCD) treated across the Lazio regional hematology network. Despite the rarity of the disease and the limited sample size, our findings support the effectiveness and tolerability of Siltuximab in routine clinical practice, with an overall response rate (ORR) of 86% and a complete remission rate (CRR) of 29%.

The demographic and clinical profile of our cohort aligns with previously published iMCD populations. The median age at diagnosis (54 years) and female predominance are consistent with data from the Castleman Disease Collaborative Network (CDCN) registry. Histopathological subtypes were heterogeneous, with plasmacytic and mixed variants predominating, while hypervascular histology accounted for 21% of cases. The high prevalence of inflammatory markers (median CRP 8.7 mg/L; ESR 45 mm/h) and B symptoms (43%) at diagnosis further underscore the systemic hyperinflammatory nature of iMCD.

The observed response rates compare favorably with the pivotal phase II study by van Rhee et al. (18) and subsequent real-world analyses (16–21). The higher ORR in our cohort may reflect broader inclusion of symptomatic and biochemical improvements typical of clinical practice, rather than strict trial criteria. When contextualized against previously published real-world datasets (**Table 2**), our findings are consistent with international experiences reporting high overall response rates and durable disease control with siltuximab in routine clinical practice, despite differences in cohort size, follow-up duration, and treatment line (21–25). The median time to best response (6 months) is consistent with the known kinetics of Siltuximab and may be influenced by the retrospective nature of the study and variability in radiologic reassessment. Importantly, 85.7% of patients were alive at the data cutoff, suggesting sustained disease control and survival benefits with continued therapy.

**Table 2:** Real-world and long-term experiences with siltuximab in idiopathic multicentric Castleman disease.

Study	Country	Study design	Patients (n)	Median follow-up (months)	Line of therapy	ORR (%)	CR (%)	Survival outcomes
Ostrowska et al., 2021	Poland	Multicenter RWD	11	53	Mixed	72.5	18	3-year OS 81%
Min et al., 2022	South Korea	Multicenter RWD	27	5	Mixed	81.5	67	5-year OS 86%
Jitaru et al., 2024	Greece/Romania	Multicenter RWD	48	47	Mixed	71	55.5	3-year OS 74%
Rossini et al., 2024	Italy	Multicenter RWD	10	—	First-line	100	—	OS not reported
Present study	Italy (Lazio region)	Multicenter RWD	14	13	First-line	86	29	OS 85.7%; EFS 64.3%

CR, complete remission; EFS, event-free survival; ORR, overall response rate; OS, overall survival; RWD, real-world data.

Two patients who were diagnosed with Hodgkin lymphoma during Siltuximab treatment subsequently received chemotherapy and remain alive at follow-up. These cases highlight the importance of careful histopathological evaluation in iMCD, as nodal progression may warrant repeat biopsy to exclude overlapping or emerging lymphoid malignancies (27).

Safety outcomes were favorable and consistent with previous reports. No infusion-related reactions occurred, and only 21% of patients experienced Grade ≥2 adverse events, including hypertension, elevated transaminases, and pneumonia. The absence of hematologic or autoimmune toxicities underscores the excellent tolerability of Siltuximab in long-term use outside of clinical trials.

This study has several limitations inherent to its retrospective design, including the small sample size and the absence of standardized quality-of-life assessments, which limit statistical power and preclude detailed subgroup analyses. However, in the context of rare diseases such as iMCD, well-characterized real-world cohorts remain highly informative. The multicenter nature of the study, strict adherence to CDCN diagnostic and response criteria, and prolonged treatment exposure achieved in several patients strengthen the clinical relevance of these findings.

Nonetheless, from a health-system perspective, the multicenter design underscores the importance of regional collaboration in the management of rare diseases such as iMCD. Integrated care pathways across academic and community hospitals facilitate standardized diagnostic work-ups, ensure equitable access to biologic therapies, and enable systematic data collection—elements that are essential for advancing epidemiologic knowledge and improving patient outcomes.

## Conclusions

In this real-world multicenter cohort, first-line Siltuximab demonstrated high activity and a favorable safety profile in patients with idiopathic Multicentric Castleman Disease. The majority of patients achieved durable disease control with minimal severe adverse events. These findings support the use of Siltuximab as a cornerstone therapy for iMCD in routine clinical practice and highlight the value of regional collaboration in managing rare hematologic disorders. Expansion of this network to a national level could further improve epidemiologic understanding, optimize patient care, and validate long-term outcomes of IL-6–targeted therapy.

## Data Availability

The original contributions presented in the study are included in the article/supplementary material. Further inquiries can be directed to the corresponding author.
