# Correction: Siltuximab as a first-line therapy for idiopathic multicentric Castleman disease: a retrospective analysis based on the SiMuLa study of the Italian regional network

**DOI:** 10.3389/fonc.2026.1812268

**Published:** 2026-03-11

**Authors:** S. Pelliccia, A. Di Rocco, G. Palumbo, A. Zizzari, O. Annibali, E. Maiolo, A. Rago, G. Trapè, M. P. Bianchi, G. Pileggi, L. Filomeno, S. Hohaus, L. Rigacci, M. C. Cox, M. Martelli, G. La Verde, A. Di Napoli, A. Tafuri

**Affiliations:** 1 Hematology, Department of Clinical and Molecular Medicine, University Hospital Sant’Andrea – Sapienza, Rome, Italy; 2 Hematology, Department of Translational and Precision Medicine Univ Sapienza, Rome, Italy; 3 Hematology, Department of Biomedicine and Prevention, University Tor Vergata, Rome, Italy; 4 Research Unit of Hematology, Department of Medicine and Surgery, Università Campus Bio- Medico di Roma, Rome, Italy; 5 University Policlinico Gemelli Foundation-IRCCS, Catholic University of the Sacred Heart, Roma, Italy; 6 Hematology Unit, Azienda Sanitaria Locale (ASL) ROMA 1, Rome, Italy; 7 Hematology Unit, Ospedale Belcolle, Viterbo, Italy; 8 Pathology Unit, Sant’Andrea Hospital, La Sapienza University, Department of Clinical and Molecular Medicine, Sapienza University, Rome, Italy

**Keywords:** Castleman disease, interleukin-6, multicentric, real-world evidence, siltuximab

In the published article, there were some errors in the references, which were erroneously written as follows:

1. Dispenzieri A, Fajgenbaum DC. Overview of Castleman disease. Blood. 2020;135(16):1353–1364. doi:10.1182/blood.2019000931

2. van Rhee F, Oksenhendler E, Srkalovic G, Voorhees P, Lim M, Casper C, et al. International evidence-based consensus diagnostic and treatment guidelines for unicentric Castleman disease. Blood Adv. 2020;4(23):6039–6050. doi:10.1182/bloodadvances.2020003048

3. Masaki Y, Arita K, Sakai T. Castleman disease and TAFRO syndrome. Ann Hematol. 2022;101(3):485–490. doi:10.1007/s00277-021-04667-2

4. Fajgenbaum DC, van Rhee F, Nabel CS. HHV-8–negative, idiopathic multicentric Castleman disease: novel insights into biology, pathogenesis, and therapy. Blood. 2014;123(19):2924–2933. doi:10.1182/blood-2013-12-545970

5. Pierson SK, Brandstadter JD, Torigian DA, Rosenbaum JN, Palmer J, Fajgenbaum DC, et al. Characterizing the heterogeneity of Castleman disease and the oligocentric subtype: findings from the ACCELERATE registry. Blood Adv. 2025;9(8):1952–1965.

6. Mukherjee S, Martin R, Sande B, Dispenzieri A, Fajgenbaum DC, van Rhee F, et al. Epidemiology and treatment patterns of idiopathic multicentric Castleman disease in the era of IL-6–directed therapy. Blood Adv. 2022;6(2):359–367. doi:10.1182/bloodadvances.2021005514

7. Masaki Y, Kawabata H, Fujimoto S, Ishigaki Y, Kurose N, Yoshizaki K, et al. Epidemiological analysis of multicentric and unicentric Castleman disease and TAFRO syndrome in Japan. J Clin Exp Hematop. 2019;59:175–181.

8. Pierson SK, Khor JS, Ziglar J, Liu A, Floess K, NaPier E et al. ACCELERATE: A Patient-Powered Natural History Study Design Enabling Clinical and Therapeutic Discoveries in a Rare Disorder. Cell Rep Med. 2020;1(9):100158. doi: 10.1016/j.xcrm.2020.100158.

9. Dispenzieri A, Armitage JO, Loe MJ, Geyer SM, Allred J, Wester SM, et al. The clinical spectrum of Castleman’s disease. Am J Hematol. 2012;87(11):997–1002. doi:10.1002/ajh.23291

10. Fajgenbaum DC, Shilling D. Castleman disease pathogenesis. Hematol Oncol Clin North Am. 2018;32(1):11–21. doi:10.1016/j.hoc.2017.09.002

11. Wu D, Lim MS, Jaffe ES. Pathology of Castleman disease. Hematol Oncol Clin North Am. 2018;32(1):37–52. doi:10.1016/j.hoc.2017.09.004

12. Pelliccia S, Rogges E, Cardoni A, Galli E, Guarini A, Foà R, et al. The application of a multidisciplinary approach in the diagnosis of Castleman disease and Castleman-like lymphadenopathies: a 20-year retrospective analysis. Br J Haematol. 2024;204(2):534–547.

13. Fajgenbaum DC, Uldrick TS, Bagg A, Frank D, Wu D, Srkalovic G, et al. International, evidence-based consensus diagnostic criteria for HHV-8–negative/idiopathic multicentric Castleman disease. Blood. 2017;129(12):1646–1657. doi:10.1182/blood-2016-10-746933

14. van Rhee F, Voorhees P, Dispenzieri A, Fosså A, Srkalovic G, Ide M, et al. International, evidence-based consensus treatment guidelines for idiopathic multicentric Castleman disease. Blood. 2018;132(20):2115–2124. doi:10.1182/blood-2018-07-862334

15. van Rhee F, Fayad L, Voorhees P, Furman R, Goy A, Casper C, et al. Siltuximab, a novel anti–interleukin-6 monoclonal antibody, for Castleman’s disease. J Clin Oncol. 2010;28(23):3701–3708. doi:10.1200/JCO.2009.27.2377

16. Kurzrock R, Voorhees PM, Casper C, Furman RR, Fayad L, Lonial S, et al. A phase I, open-label study of siltuximab in patients with B-cell non-Hodgkin lymphoma, multiple myeloma, or Castleman disease. Clin Cancer Res. 2013;19(13):3659–3670. doi:10.1158/1078-0432.CCR-13-0368

17. van Rhee F, Casper C, Voorhees PM, Fayad L, Lonial S, Ide M, et al. Long-term safety of siltuximab in idiopathic multicentric Castleman disease. Lancet Haematol. 2020;7(3):e209–e217. doi:10.1016/S2352-3026(19)30272-2

18. van Rhee F, Casper C, Voorhees PM, Fayad L, Lonial S, Ide M, et al. Long-term safety of siltuximab in multicentric Castleman disease. Oncotarget. 2015;6(30):30408–30419.

19. van Rhee F, Wong RS, Munshi N, Rossi JF, Ke XY, Fosså A, et al. Siltuximab for multicentric Castleman’s disease: a randomised, double-blind, placebo-controlled trial. Lancet Oncol. 2014;15:966–974. doi:10.1016/S1470-2045(14)70319-5

20. van Rhee F, Rosenthal A, Kanhai K, Fajgenbaum DC, Dispenzieri A, Ide M, et al. Siltuximab is associated with improved progression-free survival in idiopathic multicentric Castleman disease. Blood Adv. 2022;6:4773–4781.

21. Oksenhendler E, Boutboul D, Fajgenbaum DC, Mirouse A, Fieschi C, Malphettes M, et al. Real-world use of siltuximab in idiopathic multicentric Castleman disease. Blood. 2018;132(8):800–807. doi:10.1182/blood-2018-03-838490

22. Ostrowska B, Szymczyk A, Olszewska-Szopa M, Piszcz J, Gil L, Komarnicki M, et al. Efficacy of siltuximab in idiopathic multicentric Castleman disease: the first Polish real-world experience. Leuk Lymphoma. 2021;62(12):3031–3034.

23. Min GJ, Jeon YW, Kim TY, Kwag DH, Lee JH, Kim HJ. Long-term treatment outcome of Castleman disease: a real-world experience. Front Oncol. 2022;12:974770. doi:10.3389/fonc.2022.974770

24. Jitaru C, Miron L, Jinga D, Bogdan MA, Dumitrescu M, Popa M, et al. Siltuximab in idiopathic multicentric Castleman disease: real-world experience in Greece and Romania. J Hematol. 2024.

25. Li SY, Gao YH, Dang Y, Chang L, Zhang X, Wang J, et al. Real-world data of siltuximab for Chinese patients with idiopathic multicentric Castleman disease. Ann Hematol. 2025;104(3):1713–1720.

26. Rossini B, Cecchi N, Clemente F, Gozzetti A, Giordano L, Caracciolo F, et al. Real-practice management of idiopathic multicentric Castleman disease with siltuximab. Drugs Context. 2024;13:2023-9-4. doi:10.7573/dic.2023-9-4

27. Lyapichev KA, You MJ, Vega F, Wang SA, Medeiros LJ, Lin P, et al. Classic Hodgkin lymphoma and Castleman disease: an emerging entity. Virchows Arch. 2020;477(3):437–444. doi:10.1007/s00428-020-02831-1].

The corrected references appear below:

1. Dispenzieri A, Fajgenbaum DC. Overview of Castleman disease. *Blood*. (2020) 135:1353–64. doi:10.1182/blood.2019000931

2. van Rhee F, Oksenhendler E, Srkalovic G, Voorhees P, Lim M, Dispenzieri A, et al. International evidence-based consensus diagnostic and treatment guidelines for unicentric Castleman disease. *Blood Adv*. (2020) 4:6039–50. doi:10.1182/bloodadvances.2020003048

3. Masaki Y, Arita K, Sakai T, Takai K, Aoki S, Kawabata H. Castleman disease and TAFRO syndrome. *Ann Hematol*. (2022) 101:485–90. doi:10.1007/s00277-021-04667-2

4. Fajgenbaum DC, van Rhee F, Nabel CS. HHV-8–negative, idiopathic multicentric Castleman disease: novel insights into biology, pathogenesis, and therapy. *Blood*. (2014) 123:2924–33. doi:10.1182/blood-2013-12-545970

5. Pierson SK, Brandstadter JD, Torigian DA, Bagg A, Lechowicz MJ, Alapat D, et al. Characterizing the heterogeneity of Castleman disease and the oligocentric subtype: findings from the ACCELERATE registry. *Blood Adv*. (2025) 9:1952–65.

6. Mukherjee S, Martin R, Sande B, Paige JS, Fajgenbaum DC. Epidemiology and treatment patterns of idiopathic multicentric Castleman disease in the era of IL-6–directed therapy. *Blood Adv*. (2022) 6:359–67. doi:10.1182/bloodadvances.2021005514

7. Masaki Y, Kawabata H, Fujimoto S, Kawano M, Iwaki N, Kotani T, et al. Epidemiological analysis of multicentric and unicentric Castleman disease and TAFRO syndrome in Japan. *J Clin Exp Hematop*. (2019) 59:175–81.

8. Pierson SK, Khor JS, Ziglar J, Liu A, Floess K, NaPier E et al. ACCELERATE: A Patient-Powered Natural History Study Design Enabling Clinical and Therapeutic Discoveries in a Rare Disorder. *Cell Rep Med*. (2020) 1:100158. doi: 10.1016/j.xcrm.2020.100158.

9. Dispenzieri A, Armitage JO, Loe MJ, Geyer SM, Allred J, Camoriano JK et al. The clinical spectrum of Castleman’s disease. *Am J Hematol*. (2012) 87:997–1002. doi:10.1002/ajh.23291

10. Fajgenbaum DC, Shilling D. Castleman disease pathogenesis. *Hematol Oncol Clin North Am*. (2018) 32:11–21. doi:10.1016/j.hoc.2017.09.002

11. Wu D, Lim MS, Jaffe ES. Pathology of Castleman disease. *Hematol Oncol Clin North Am*. (2018) 3237–52. doi:10.1016/j.hoc.2017.09.004

12. Pelliccia S, Rogges E, Cardoni A, Lopez G, Conte E, Faccini AL, et al. The application of a multidisciplinary approach in the diagnosis of Castleman disease and Castleman-like lymphadenopathies: a 20-year retrospective analysis. *Br J Haematol*. (2024) 204:534–47.

13. Fajgenbaum DC, Uldrick TS, Bagg A, Frank D, Wu D, Srkalovic G, et al. International, evidence-based consensus diagnostic criteria for HHV-8–negative/idiopathic multicentric Castleman disease. *Blood*. (2017) 129:1646–57. doi:10.1182/blood-2016-10-746933

14. van Rhee F, Voorhees P, Dispenzieri A, Fosså A, Srkalovic G, Ide M, et al. International, evidence-based consensus treatment guidelines for idiopathic multicentric Castleman disease. *Blood*. (2018) 132:2115–24. doi:10.1182/blood-2018-07-862334

15. van Rhee F, Fayad L, Voorhees P, Lonial S, Borghaei H, et al. Siltuximab, a novel anti–interleukin-6 monoclonal antibody, for Castleman’s disease. *J Clin Oncol*. (2010) 28:3701–8. doi:10.1200/JCO.2009.27.2377

16. Kurzrock R, Voorhees PM, Casper C, Furman RR, Fayad L, Lonial S, et al. A phase I, open-label study of siltuximab in patients with B-cell non-Hodgkin lymphoma, multiple myeloma, or Castleman disease. *Clin Cancer Res*. (2013) 19:3659–70. doi:10.1158/1078-0432.CCR-13-0368

17. van Rhee F, Casper C, Voorhees PM, Fayad L, Gibson D, Kanhai K, et al. Long-term safety of siltuximab in idiopathic multicentric Castleman disease. *Lancet Haematol*. (2020) 7:e209–17. doi:10.1016/S2352-3026(19)30272-2

18. van Rhee F, Casper C, Voorhees PM, Fayad L, van de Velde H, Vermeulenet J al. A phase 2, open-label, multicenter study of the long-term safety of siltuximab (an anti-interleukin-6 monoclonal antibody) in patients with multicentric Castleman disease. *Oncotarget*. (2015) 6:30408–19. doi: 10.18632/oncotarget.4655.

19. van Rhee F, Wong RS, Munshi N, Rossi JF, Ke XY, Fosså A, et al. Siltuximab for multicentric Castleman’s disease: a randomised, double-blind, placebo-controlled trial. *Lancet Oncol*. (2014) 15:966–74. doi:10.1016/S1470-2045(14)70319-5

20. van Rhee F, Rosenthal A, Kanhai K, Fajgenbaum DC, Dispenzieri A, Ide M, et al. Siltuximab is associated with improved progression-free survival in idiopathic multicentric Castleman disease. *Blood Adv*. (2022) 6:4773–81.

21. Oksenhendler E, Boutboul D, Fajgenbaum DC, Mirouse A, Fieschi C, Malphettes M, et al. The full spectrum of Castleman disease: 273 patients studied over 20 years. *Br J Haematol*. (2018) 180:206–16. doi: 10.1111/bjh.15019

22. Ostrowska B, Szymczyk A, Olszewska-Szopa M, Piszcz J, Gil L, Komarnicki M, et al. Efficacy of siltuximab in the treatment of idiopathic multicentric castleman disease, the first Polish, real-world experience with long-term observation. *Leuk Lymphoma*. (2021) 62:3031–34. doi: 10.1080/10428194.2021.1941926.

23. Min GJ, Jeon YW, Kim TY, Kwag DH, Lee JH, Lee JY. Long-term treatment outcome of Castleman disease: a real-world experience. *Front Oncol*. (2022) 12:974770. doi:10.3389/fonc.2022.974770

24. Jitaru C, Symeonidis A, Badelita S, Katodritou E, Colita A, Mpanti A et al. Siltuximab in Idiopathic Multicentric Castleman Disease: Real-World Experience. *J Hematol*. (2024) 13:207–15. doi: 10.14740/jh1343

25. Li SY, Gao YH, Dang Y, Chang L, Zhang X, Wang J, et al. Real-world data of siltuximab for Chinese patients with iMCD: combination with BCD regimen as a potential approach for severe cases. *Ann Hematol*. (2025) 104:1713–20. doi: 10.1007/s00277-025-06329-7

26. Rossini B, Cecchi N, Clemente F, De Paolis MR, Hohaus S, Innao V, et al. Real-practice management and treatment of idiopathic multicentric Castleman disease with siltuximab: a collection of clinical experiences. *Drugs Context*. (2024) 13:2023-9-4. doi:10.7573/dic.2023-9-4

27. Lyapichev KA, You MJ, Vega F, Solis LM, Medeiros LJ. Classic Hodgkin lymphoma and Castleman disease: an entity appears to be emerging *Virchows Arch*. (2020) 477:437–44. doi:10.1007/s00428-020-02831-1].

There was a mistake in **Table 2** as published. The data reported in the table were inaccurate, and the first row has therefore been removed.

**Table 2:** Real-world and long-term experiences with siltuximab in idiopathic multicentric Castleman disease.

Study	Country	Study design	Patients (n)	Median follow-up (months)	Line of therapy	ORR (%)	CR (%)	Survival outcomes
Ostrowska et al., 2021	Poland	Multicenter RWD	11	53	Mixed	72.5	18	3-year OS 81%
Min et al., 2022	South Korea	Multicenter RWD	27	53	Mixed	81.5	67	5-year OS 86%
Jitaru et al., 2024	Greece/Romania	Multicenter RWD	48	47	Mixed	71	55.5	3-year OS 74%
Rossini et al., 2024	Italy	Multicenter RWD	10	_	First-line	100	–	OS not reported
Present study	Italy (Lazio region)	Multicenter RWD	14	13	First-line	86	29	OS 85.7%; EFS 64.3%

CR, complete remission; EFS, event-free survival; ORR, overall response rate; OS, overall survival; RWD, real-world data.

The corrected **Table 2** appears below.

The original version of this article has been updated.

